# Contraceptive use pattern based on the number and composition of children among married women in sub-Saharan Africa: a multilevel analysis

**DOI:** 10.1186/s40834-023-00240-0

**Published:** 2023-07-24

**Authors:** Achamyeleh Birhanu Teshale, Vicky Qi Wang, Godness Kye Biney, Edward Kwabena Ameyaw, Nicholas Kofi Adjei, Sanni Yaya

**Affiliations:** 1 Department of Epidemiology and Biostatistics, Institute of Public Health, College of Medicine and Health Sciences, University of Gondar, Gondar, Ethiopia; 2 School of Public Health and Preventive Medicine, Monash University, Melbourne, VIC Australia; 3 Institute of Policy Studies and School of Graduate Studies, Lingnan University, Tuen Mun, Hong Kong; 4 Department of Biostatistics and Epidemiology, School of Public Health and Health Sciences, University of Massachusetts, Amherst, USA; 5 L & E Research Consult Ltd, Upper West Region, Ghana; 6 Department of Public Health and Policy, University of Liverpool, Liverpool, UK; 7 School of International Development and Global Studies, University of Ottawa, Ottawa, ON Canada; 8 The George Institute for Global Health, Imperial College London, London, UK

**Keywords:** Contraception, Reproductive health, Children, Women, Sexual health, Global health, Sub-saharan Africa

## Abstract

**Background:**

The relationship between composition of children and contraception use has received limited scholarly attention in sub-Saharan Africa. In this study, we examined the relationship between contraceptive methods, the number and composition of children in SSA.

**Methods:**

Data on 21 countries in sub-Saharan Africa (SSA) countries that had a Demographic and Health Survey on or before 2015 were analysed. We applied a multilevel multinomial logistic regression model to assess the influence of family composition on contraceptive use. Adjusted relative risk ratio (aRRR) and 95% CI were estimated. The significant level was set at p < 0.05. All the analyses were conducted using weighted data.

**Results:**

Women who had one son and two daughters (aRRR = 0.85, CI = 0.75, 0.95), two sons and one daughter (aRRR = 0.81 CI = 0.72, 0.92), one son and three daughters (aRRR = 0.66, CI = 0.54, 0.80), two sons and two daughters (aRRR = 0.59, CI = 0.50, 0.69), and three or more sons (aRRR = 0.75, CI = 0.63, 0.91) were less likely to use temporary modern contraceptive methods. Those with two sons and two daughters were less likely to use traditional methods (aRRR = 0.52, CI = 0.35, 0.78). Women in the older age group (35–49 years) were less likely to use temporary modern methods (aRRR = 0.60; 95%CI; 0.57, 0.63). However, this group of women were more likely to use permanent (sterilization) (aRRR = 1.71; 95%CI; 1.50, 1.91) and traditional methods (aRRR = 1.28; 95%CI; 1.14, 1.43).

**Conclusion:**

These findings suggest that contraception needs of women vary based on the composition of their children, hence a common approach or intervention will not fit. As a result, contraception interventions ought to be streamlined to meet the needs of different categories of women. The findings can inform policymakers and public health professionals in developing effective strategies to improve contraceptive use in SSA.

## Background

Contraceptive methods and family planning knowledge are essential components of reproductive health, as they enable individuals to make informed decisions regarding their fertility and to plan their families based on their desired timing and spacing of children [1]. Contraception is also a crucial public health intervention that can help prevent unintended pregnancies and reduce maternal and infant morbidity and mortality [2]. However, empirical studies in sub-Saharan Africa (SSA) indicate that the actual use of contraception remains suboptimal, with limited impact on fertility rates [3, 4]. However, the contraceptive utilisation rate in SSA (22-33%) is comparable to that of other developing countries like Timor-Leste and Afghanistan (i.e. 29%) [5]. Understanding the factors that influence contraceptive use is vital in developing effective interventions to promote contraceptive methods and improve reproductive health outcomes [6].

Individual factors such as women’s age, education, income, parity, and knowledge of contraception have been found to be associated with contraceptive utilization in SSA [7, 8]. Several studies have demonstrated, for instance, that women in SSA with higher levels of education and income are more likely to use contraception because they have greater control over their reproductive lives and greater access to information and services [8–11]. Regarding social factors, gender norms may also play a role, as some women may lack the autonomy to make decisions regarding their reproductive health without the consent of their partners or families [12]. It has been demonstrated that partner support is a significant predictor of contraceptive use, as women with supportive partners are more likely to use contraception [6]. Another important factor is media exposure. A systematic review and meta-analysis of 47 studies demonstrated a significant positive effect of media exposure on contraceptive use after adjusting for confounders [13].

In addition to these factors, the number of children [8], and preference for sons [14] have been associated with contraceptive use. Studies have shown that women with no or one child are less likely to use contraception, possibly because they desire more children or do not perceive the need for contraception at that time. Ahinkorah et al. have also noted that women with four or more children are more likely to use contraception because they may feel that their family is complete and wish to prevent unintended pregnancies [15].

Compared to women with first-born sons, women with first-born daughters are less likely to use contraceptives [14]. This could be attributed to the social and cultural stigma related to giving birth to daughters, leading women to expect children in the hopes of having a son [16]. Moreover, it is not uncommon for husbands to have preference for sons and, therefore, discourage their partners from using contraception, especially if they believe it will decrease their chances of having a son [17]. While researchers have examined the associations between the number of children and son preference, further studies are required to investigate the influence of interactions between the number of children, children composition and type of contraception (such as long-term and short-term methods), as this aspect has not received sufficient scholarly attention in the SSA literature. This study contributes to expanding existing knowledge by investigating the relationships between women’s contraceptive choices, the number of children, composition of children and other critical socio-demographic characteristics of SSA women.

## Methods

### Data source

The study included sub-Saharan Africa (SSA) countries that had Demographic and Health Surveys from 2015 to the present. In total, 22 countries met this criterion. However, Burkina Faso was excluded because it lacked data on contraceptive use even though its most recent DHS was conducted between 2017 and 2018. Consequently, 21 countries were included (see Table 1). The primary demographic and health indicators were gathered in each DHS, and all of these surveys used a stratified two-stage cluster sampling technique. The study population comprised married reproductive-age women who had decided not to have any more children. Pregnant women, infertile women, those who did not have at least one child at the time of the survey, and women with missing data about contraceptive use were excluded. Finally, a total weighted sample of 48,184 (unweighted sample = 48,699) was used for this study. Table 1 presents a list of countries with their respective sample size (both weighted and unweighted) and the survey year/period.

**Table 1:** List of countries included, sample size, and survey year/period

Country (year)	Unweighted sample (n = 48,699)	Percentage (%)	Weighted sample (49,184)	Percentage (%)
Angola (2015/16)	463	0.95	460	0.94
Benin (2017/18)	1,979	4.06	1,939	3.94
Burundi (2016/17)	3,019	6.2	3,038	6.18
Cameroon (2018)	1,390	2.85	1,382	2.81
Ethiopia (2016)	2,586	5.31	3,335	6.78
Gambia (2019/20)	1,297	2.66	1,188	2.41
Guinea (2018)	1,293	2.66	1,255	2.55
Liberia (2019/20)	920	1.89	764	1.55
Madagascar (2021)	3,475	7.14	3,615	7.35
Malawi (2015/16)	1,485	3.05	1,603	3.26
Mali (2018)	1,674	3.44	1,600	3.25
Mauritania (2019/20)	6,803	13.97	6,934	14.10
Nigeria (2018)	6,604	13.56	6,434	13.08
Rwanda (2019/20)	2,430	4.99	2,411	4.90
Senegal (2019)	2,365	4.86	2,286	4.65
Sierra Leone (2019)	1,000	2.05	1,026	2.09
Tanzania (2015/16)	1,640	3.37	1,698	3.45
Uganda (2016)	2,304	4.73	2,211	4.49
South Africa (2016)	1,122	2.3	1,184	2.41
Zambia (2018)	2,598	5.33	2,584	5.25
Zimbabwe (2015)	2,252	4.62	2,237	4.55

### Study variables

#### Outcome variable

The outcome variable was the current use of contraception, which was categorized as none, temporary modern method, permanent methods (male or female sterilisation), or traditional methods. This categorisation is based on the DHs Guideline (https://dhsprogram.com/data/Guide-to-DHS-Statistics/index.cfm).

### Independent variable

The main independent variable was family composition (number and sex of living children). The number of living children was assumed to be from the current marriage. This variable was categorised into nine: (1) one or two sons only; (2) two: one son and one daughter; (3) three: one son and two daughters; (4) three: two sons and one daughter; (5) four: one son and three daughters; (6) four: two sons and two daughters; (7) three or more sons only; (8) at least one daughter; and (9) others. These categories were used in previous studies [18, 19].

### Covariates

Based on the availability of suitable variables in the DHS data and previous studies [11, 20, 21], nine variables were included as covariates. These variables were: age (categorised as 15–34 and 35–49), wealth status (poorest, poorer, middle, richer, and richest), the respondent/women’s education (no formal education, primary, secondary, and higher education) and husband education (no formal education, primary, secondary, and higher education). Other covariates included working status (working and not working), sex of household head (male and female), residence (urban and rural), media exposure (no exposure, exposure to one media, exposure to two media, and exposure to three media), and participating in household decisions (yes and no).

Media exposure referred to exposure to family planning messages in the last few months through radio, television, and/or newspaper/magazine. These exposure variables were combined and categorised as: no media exposure, exposure to at least one media, exposure to two media, and exposure to three media. Participating in household decisions was also a composite variable generated from three questions regarding who decides on respondents’ health care, major household purchases, and visits to family or relatives. If women made decisions themselves, they were considered to be participating in household decision-making [22].

### Statistical analysis

Background characteristics were reported using frequencies and percentages. The proportion of contraceptive use and its 95% confidence interval (CI) were reported. To assess the influence of family composition on contraceptive use, a multilevel multinomial (mixed effect) logistic regression model was applied using STATA “*gsem”* command. We employed a multilevel multinomial logistic regression model due to the hierarchical nature of the DHS data and the presence of multiple categories in the outcome variable. We fitted four models; the null model (without any explanatory variables), model 1 (containing only the individual level variables), model 2 (using a community level factor variable, residence), and model 3 (the final model containing both individual and community level variables). However, we reported the two models; the null model just to assess the clustering or community level variability of contraceptive use via the intraclass correlation coefficient (ICC) and the final model (model 3) since it was the best fitted model (had highest loglikelihood and lowest deviance). Before performing the multivariable analysis (i.e. the fixed effects analysis) which incorporated the covariates and the independent variable, a machine learning approach called Least Absolute Shrinkage and Selection Operator (LASSO) regression was applied for variable selection [23]. Finally, we fitted a multivariable multilevel multinomial logistic regression and adjusted relative risk ratio (aRRR) and their 95%CI were reported. The random effect analysis was used to calculate the random effect parameters (ICC and variance) of the null model. All the analyses were conducted using the weighted sample. Stata 17 was used for the analysis and statistical significance was declared at p < 0.05.

### Ethical consideration

#### Ethical approval

was not required for this study as the data used were secondary and available in the public domain anonymously.

## Results

### Background characteristics

Table 2 presents the background characteristics of the study participants. The majority of women (71.52%) were in the age group 35 and above. Approximately 85.47% of women were from male-headed households, while only 7.15% of women participated in household decisions, solely responsible for their health care, household purchase, and visiting family. More than half of women had no exposure to media (i.e., not exposed to family planning messages from at least one media in the last few months. More than two-thirds (67.70%) of women resided in rural areas. In terms of family composition, the most common was “other” family composition (49.09%), followed by at least one daughter but no son (16.61%) and one or two sons only (12.16%). Regarding the educational level of women and their husbands, the majority had no formal education and had primary education (Table 2).

**Table 2:** Background characteristics

Characteristics	Unweighted frequency (n = 48,699)	Percentage (%)	Weighted frequency (n = 49,184)	Percentage (%)
Age (years) 15–34 35–49	13,577 35,122	27.88 72.12	14,006 35,178	28.48 71.52
Wealth Poorest Poorer Middle Richer Richer	8,799 9,243 9,942 9,981 10,734	18.07 18.98 20.42 20.50 22.04	8,275 9,346 9,982 10,379 11,202	16.82 19.00 20.30 21.10 22.77
Respondent education No formal education Primary Secondary Higher	18,290 18,442 9,816 2,151	37.56 37.87 20.16 4.42	18,549 18,636 9,788 2,211	37.71 37.89 19.90 4.50
Husband education No formal education Primary Secondary Higher	15,804 16,741 12,210 3,944	32.45 34.38 25.07 8.10	15,940 17,229 12,113 3,902	32.41 35.03 24.63 7.93
Currently working/occupation No Yes	13,362 35,337	27.44 72.56	13,695 35,489	27.84 72.16
Sex of household head Male Female	41,393 7,306	85.00 15.00	42,036 7,148	85.47 14.53
Residence Urban Rural	15,698 33,001	32.23 67.77	15,885 33,299	32.30 67.70
Media exposure Not Exposed Exposed to one media Exposed to two media Exposed to all media	27,320 13,658 5,567 2,154	56.10 28.05 11.43 4.42	27,467 13,799 5,755 2,163	55.85 28.06 11.70 4.40
Number and sex of living children One or two: son(s) only Two: one son, one daughter Three: one son, two daughters Three: two sons, one daughter Four: one son, two daughters Four: two sons, two daughters Three or more sons At least one daughter Other	6,126 3,407 1,940 1,691 757 1,047 724 9,287 23,720	12.58 7.00 3.98 3.47 1.55 2.15 1.49 19.07 48.71	6,202 3,408 1,898 1,632 744 1,026 707 9,422 24,145	12.61 6.93 3.84 3.32 1.15 2.09 1.44 19.16 49.09
Women solely decide Household decisions No Yes	45,157 3,542	92.73 7.27	45,665 3,519	92.85 7.15

### Prevalence of contraceptive use in SSA

More than half, 53.15% (95%CI; 52.71%, 53.59%) of the women did not use contraceptives, despite wanting no more children. Among the users, temporary modern methods were commonly used, 36.15% [95%CI; 35.73%, 36.58%] (Table 3).

**Table 3:** Contraceptive use in SSA

Contraceptive use	Frequency	Percentage (95%CI)
Do not use any method	26,140.987	53.15 (52.71, 53.59)
Temporary methods (modern)	17,781.706	36.15 (35.73, 36.58)
Permanent methods (modern)	3,111.5456	6.33 (6.11, 6.54)
Traditional methods	2,149.6497	4.37 (4.19, 4.55)

### Multilevel multinomial logistic regression modeling

#### Random effect analysis:

This model was used to indicate community/cluster level variability in the occurrence of the outcome variable. The variance (and standard error) and the ICC of the null model were 0.174 (0.014) and 5% respectively. The ICC and variance were decreased in the final model (Table 4). The random effect parameters, especially in the null model, revealed the need to account for cluster level variability.

#### Fixed effect analysis (assessment of factors associated with contraceptive use):

Eligible variables for the multivariable multilevel multinomial regression were selected using LASSO. In LASSO, the 64th model was determined to be the most parsimonious, with an optimum lambda (penalty factor) of 0.0002 and the lowest cross-validation mean deviance. All variables had different coefficients from zero and were eligible for the multivariable multilevel analysis.

Family composition: Women who had one son and two daughters, two sons and one daughter, one son and three daughters, two sons and two daughters, and three or more sons were less likely to use temporary modern contraceptive methods. Women with two sons and two daughters were less likely to use traditional methods. However, women with one or two sons only, one son and daughter, one son and two daughters, one son and three daughters, and at least one daughter were more likely to use permanent contraceptive methods.

Table 4 presented the significant covariates. Of these, older age group (35–49 years) women were less likely to use temporary modern methods (aRRR = 0.60; 95%CI; 0.57, 0.63). However, this group of women were more likely to use permanent (sterilization) (aRRR = 1.71; 95%CI; 1.50, 1.91) and traditional methods (aRRR = 1.28; 95%CI; 1.14, 1.43). Women from wealthy households had a higher likelihood of using all types of contraceptive methods (temporary modern, permanent [sterilization], and traditional methods). The strength of this association increased as wealth status improved, from poorer to richest. Being educated, both for the respondent and their partner, was associated with a higher likelihood of using all types of contraceptives. The strength of this likelihood increased as the educational level progressed from primary to higher education.

Currently working women had a higher likelihood of using permanent (aRRR = 1.19; 95%CI; 1.08, 1.30) and traditional methods (aRRR = 1.83; 95%CI; 1.62, 2.07), but not temporary modern methods (aRRR = 0.98; 95%CI; 0.93, 1.02). Women from female-headed households, were less likely to use all three contraceptive methods. Women from rural areas were more likely to use the three contraceptive methods. Women who had been exposed to the media family planning messages in the last month via one media, two media, and all three media were more likely to use all three contraceptive methods. Furthermore, women who made household decisions alone were less likely to use all three contraceptive methods (Table 4).

**Table 4:** Multivariable multilevel multinomial logistic regression analysis for factors associated with contraceptive use in sub-Saharan Africa

Characteristics	Modern (temporary) method vs. no use: aRRR (95%CI)	Modern (permanent/sterilization) method vs. no use: aRRR (95%CI)	Traditional method vs. no use: aRRR (95%CI)
Age (years) 15–34 35–49	1.00 0.60 (0.57, 0.63) ***	1.00 1.71 (1.5, 1.91) ***	1.00 1.28 (1.14, 1.43) ***
Number and sex of living children One or two: son(s) only Two: one son, one daughter Three: one son, two daughters Three: two sons, one daughter Four: one son, three daughters Four: two sons, two daughters Three or more sons At least one daughter Other	0.99 (0.92, 1.05) 0.96 (0.88, 1.05) 0.85 (0.75, 0.95) ** 0.81 (0.72, 0.92) ** 0.66 (0.54, 0.80) *** 0.59 (0.50, 0.69) *** 0.75 (0.63, 0.91) ** 1.03 (0.98, 1.09) 1.00	1.13 (1.00, 1.28) * 1.30 (1.12, 1.51) ** 1.44 (1.20, 1.72) *** 1.07 (0.86, 1.32) 1.69 (1.3, 2.18) *** 1.02 (0.78, 1.31) 1.09 (0.80, 1.49) 1.20 (1.08, 1.33) ** 1.00	0.97 (0.84, 1.12) 0.89 (0.73, 1.08) 0.82 (0.64, 1.06) 0.76 (0.58, 1.01) 0.80 (0.54, 1.20) 0.52 (0.35, 0.78) * 0.71 (0.46, 1.09) 0.92 (0.81, 1.04) 1.00
Wealth Poorest Poorer Middle Richer Richer	1.00 1.09 (1.02, 1.17) * 1.26 (1.18, 1.35) *** 1.33 (1.24, 1.44) *** 1.32 (1.21, 1.44) ***	1.00 1.14 (0.98, 1.32) 1.40 (1.21, 1.61) *** 1.82 (1.58, 2.10) *** 2.49 (2.12, 2.92) ***	1.00 1.28 (1.05, 1.57) * 1.59 (1.31, 1.93) *** 2.70 (1.71, 2.50) *** 2.68 (2.19, 3.28) ***
Respondent education No formal education Primary Secondary Higher	1.00 2.10 (1.99, 2.22) *** 2.54 (2.36, 2.73) *** 3.13 (2.76, 3.56) ***	1.00 2.32 (2.10, 2.57) *** 1.81 (1.57, 2.09) *** 2.69 (2.15, 3.38) ***	1.00 2.50 (2.18, 2.86) *** 3.36 (2.86, 3.96) *** 4.44 (3.49, 5.64) ***
Husband education No formal education Primary Secondary Higher	1.00 1.87 (1.77, 1.98) *** 1.72 (1.6, 1.83) *** 1.42 (1.28, 1.57) ***	1.00 3.57 (3.16, 4.02) *** 2.16 (1.87, 2.49) *** 2.34 (1.91, 2.84) ***	1,00 2.00 (1.74, 2.30) *** 1.48 (1.27, 1.74) *** 1.23 (1.00, 1.53) *
Currently working/occupation No Yes	1.00 0.98 (0.93, 1.02)	1.00 1.19 (1.08, 1.30) ***	1.00 1.83 (1.62, 2.07) ***
Sex of household head Male Female	1.00 0.78 (0.74, 0.83) ***	1.00 0.66 (0.59, 0.75) ***	1.00 0.67 (0.58, 0.77) ***
Residence Urban Rural	1.00 1.36 (1.29, 1.44) ***	1.00 1.97 (1.77, 2.19) ***	1.00 1.21 (1.08, 1.35) ***
Media exposure Not Exposed Exposed to one media Exposed to two media Exposed to all media	1.00 1.29 (1.228424 1.35) 1.23 (1.14, 1.31) 1.27 (1.14, 1.44)	1.00 1.41 (1.29, 1.53) *** 1.11 (0.97, 1.26) 1.53 (1.27, 1.84) ***	1.00 1.53 (1.38, 1.70) *** 1.44 (1.25, 1.65) *** 1.31 (1.07, 1.60) ***
Women solely decide Household decisions No Yes	1.00 0.86 (0.79, 0.93) ***	1.00 0.86 (0.74, 1.01)	1.00 0.75 (0.62, 0.91) **
Random effect model			
Variance (standard error) of the null model	0.174 (0.015)		
ICC of the null model	0.050		
Variance (standard error) of the final model	0.0659(0.009)		
ICC of the final model	0.020		

***; p < 0.001, **; p < 0.01, *; p < 0.05

**Abbreviations**: aRRR; adjusted relative risk ratio, CI; Confidence Interval

## Discussion

The low prevalence of contraceptive use in SSA is a major concern. This is because it is associated with unmet need for contraception and unintended pregnancies thereby to higher tendencies of adverse pregnancy outcomes [24]. In this study, we investigated the association between the composition of children and the type of contraception used. We found that more than half of the women did not use contraceptives, and among those who did, temporary modern methods were the most commonly used. This finding is consistent with previous studies that have shown low rates of contraceptive use in SSA due to various factors, including cultural and religious beliefs, limited access to family planning services, and inadequate knowledge about contraceptives [9, 25–27].

Our study revealed that one of the important driving factors influencing women’s preference for modern contraception use, as opposed to non-use and traditional methods is the number and composition of children. Thus, women with specific composition of children, such as having one son and two daughters, or two sons and one daughter were less likely to use temporary modern contraceptive methods. This finding is consistent with previous studies that have shown that gender preference and son preference can influence contraceptive use in certain societies [7, 14, 16, 20]. Furthermore, women in the older age group (35–49 years) were less inclined to use temporary modern methods but showed a higher likelihood of using permanent and traditional methods. This could be because older women may have already attained their desired number of children and therefore prefer permanent contraception methods. This finding aligns with previous studies that have established a significant association between older age and contraception use [20, 28, 29]. Nonetheless, this finding contradicts the results of a study conducted in Ghana within the SSA region by Agyemang et al. [3], which reported no significant association between age and other sociodemographic variables with contraception use [3].

Furthermore, we showed that working-class women were more likely to use permanent and traditional methods but not temporary modern methods. This may be attributed to several reasons, such as fear of potential side effects hindering their ability to work effectively, cultural beliefs or perceptions favouring permanent and traditional methods, and challenges to accessing and adherence to temporal methods [30]. This finding coincides with a study conducted in India by McDougal et al. which revealed that some women in the working class utilize traditional and permanent methods more than temporary methods [31]. One sub-Saharan African study offered a plausible explanation by asserting that women generally opt for specific contraception methods based on their reproductive health circumstances and choose traditional methods when they want to use less effective forms of contraception [29].

Wealth and education status were also found to be significant predictors of contraceptive use, with women from wealthier households and those with higher education levels having a higher likelihood of using all types of contraceptives. These findings are consistent with previous research that has shown a substantial association between maternal socioeconomic status, such as education and wealth index, and contraception use [20, 32]. Education can increase knowledge about contraceptives and family planning, empowering women to make informed decisions about their reproductive health. However, these findings contradict a study conducted by Emina et al. [9] who reported that an increase in the proportion of women with secondary education does not explain the change in contraceptive use in most SSA countries, as well as other studies that have found no relationship between the level of education and contraception use [33]. The findings indicate that educated women are more likely to be well informed about the benefits of using contraception. Besides, when women are pursuing their education dreams, they are likely to postpone or space childbearing and as a result have a relatively increased need for contraception. This might account for why educated women had higher likelihood of contraception use. With respect to wealth, women with higher wealth standing have the purchasing power and stand a greater chance of procuring any contraception they desire to use compared to the poor [34–36]. As a result, it is not surprising that wealthier women had increased likelihood of contraception use.

Women from female-headed households and those who made household decisions alone were less likely to use all three contraceptive methods. This could be attributed to several reasons, such as lack of partner support, stigma and discrimination related to their status. These factors are influenced by cultural beliefs while the social context of the SSA region could also potentially impact the accessibility and use of contraception among these women [6]. Media exposure was also a significant predictor of contraceptive use. Women who had been exposed to family planning messages via one or more media in the last month were more likely to use traditional contraceptive methods. This finding is consistent with previous studies that have shown a positive association between media exposure and contraception use [13]. However, this finding is inconsistent with a study by Ahmed et al., which reported that women exposed to mass media family planning messages are less likely to use modern contraception [37]. This finding suggests that increasing media campaigns and education on family planning and contraceptive use can improve knowledge and increase the uptake of contraceptives. Education and media campaigns can be effective in increasing knowledge and changing attitudes toward contraceptives. Moreover, increasing access to family planning services and addressing cultural and religious beliefs that may hinder contraceptive use can also improve uptake. Additionally, targeting specific groups, such as women with certain family compositions, can help increase the uptake of contraceptives in these populations.

### Strengths and limitations

We used rigorous and appropriate analytical techniques in our analysis, thereby producing robust results. Thirdly, due to the representative sampling approach, our findings, conclusions, and recommendations are generalisable to reproductive-aged women in the included countries. Despite the strengths, the study is not devoid of limitations. First, a cross-sectional study design was employed; hence causal inference between contraception and the composition of children cannot be drawn. Additionally, there is the possibility that social desirability bias might have affected some responses regarding the type of contraception used.

## Conclusion

The study indicates that the specific number and composition of children, such as having one son and two daughters, or two sons and one daughter affect contraceptive choice. Women with at least one son have a lower likelihood of using temporary contraceptive methods. Meanwhile, they typically tend to have a higher likelihood of using permanent/sterilization methods. Those with at least one daughter were also more likely to use permanent contraceptive methods. These findings suggest that contraceptive needs of women vary based on the composition of their children, hence a common approach or intervention will not fit. As a result, contraception interventions ought to be streamlined to meet the needs of different categories of women. Our findings can inform policymakers and public health professionals in developing effective strategies to enhance contraceptive use in SSA. Qualitative research may be required to unravel the rationale for the observed patterns this may offer further insights to guide practice.

## Data Availability

Data for this study were sourced from Demographic and Health surveys (DHS) and available here: http://dhsprogram.com/data/available-datasets.cfm.
